# Prevalence and phylogenetic analysis of hepatitis E virus in pigs, wild boars, roe deer, red deer and moose in Lithuania

**DOI:** 10.1186/s13028-018-0367-7

**Published:** 2018-02-23

**Authors:** Ugne Spancerniene, Juozas Grigas, Jurate Buitkuviene, Judita Zymantiene, Vida Juozaitiene, Milda Stankeviciute, Dainius Razukevicius, Dainius Zienius, Arunas Stankevicius

**Affiliations:** 1 Department of Anatomy and Physiology, Faculty of Veterinary Medicine, Lithuanian University of Health Sciences, Tilzes str. 18, Kaunas, Lithuania; 2 National Food and Veterinary Risk Assessment Institute, J. Kairiukscio str. 10, Vilnius, Lithuania; 3 Department of Animal Breeding and Nutrition, Faculty of Animal Husbandry Technology, Lithuanian University of Health Sciences, Tilzes str. 18, Kaunas, Lithuania; 4 Faculty of Medicine, Lithuanian University of Health Sciences, A. Mickeviciaus str. 9, Kaunas, Lithuania; 5 Faculty of Veterinary Medicine, Institute of Microbiology and Virology, Lithuanian University of Health Sciences, Tilzes str. 18, Kaunas, Lithuania

**Keywords:** Moose, ORF2, Phylogenetic analysis, Pig, Red deer, Roe deer, Wild boar

## Abstract

**Background:**

Hepatitis E virus (HEV) is one of the major causes of acute viral hepatitis worldwide. In Europe, food-borne zoonotic transmission of HEV genotype 3 has been associated with domestic pigs and wild boar. Controversial data are available on the circulation of the virus in animals that are used for human consumption, and to date, no gold standard has yet been defined for the diagnosis of HEV-associated hepatitis. To investigate the current HEV infection status in Lithuanian pigs and wild ungulates, the presence of viral RNA was analyzed by nested reverse transcription polymerase chain reaction (RT-nPCR) in randomly selected samples, and the viral RNA was subsequently genotyped.

**Results:**

In total, 32.98 and 22.55% of the domestic pig samples were HEV-positive using RT-nPCR targeting the ORF1 and ORF2 fragments, respectively. Among ungulates, 25.94% of the wild boar samples, 22.58% of the roe deer samples, 6.67% of the red deer samples and 7.69% of the moose samples were positive for HEV RNA using primers targeting the ORF1 fragment. Using primers targeting the ORF2 fragment of the HEV genome, viral RNA was only detected in 17.03% of the wild boar samples and 12.90% of the roe deer samples. Phylogenetic analysis based on a 348-nucleotide-long region of the HEV ORF2 showed that all obtained sequences detected in Lithuanian domestic pigs and wildlife belonged to genotype 3. In this study, the sequences identified from pigs, wild boars and roe deer clustered within the 3i subtype reference sequences from the GenBank database. The sequences obtained from pig farms located in two different counties of Lithuania were of the HEV 3f subtype. The wild boar sequences clustered within subtypes 3i and 3h, clearly indicating that wild boars can harbor additional subtypes of HEV. For the first time, the ORF2 nucleotide sequences obtained from roe deer proved that HEV subtype 3i can be found in a novel host.

**Conclusion:**

The results of the viral prevalence and phylogenetic analyses clearly demonstrated viral infection in Lithuanian pigs and wild ungulates, thus highlighting a significant concern for zoonotic virus transmission through both the food chain and direct contact with animals. Unexpected HEV genotype 3 subtype diversity in Lithuania and neighboring countries revealed that further studies are necessary to understand the mode of HEV transmission between animals and humans in the Baltic States region.

## Background

Hepatitis E virus (HEV) which causes a food and water borne disease in humans [1], has emerged during the past decade as a causative agent of autochthonous hepatitis in developed countries [2]. Meat and meat-derived products from HEV-infected reservoir animals can transmit the virus to humans and represent a public health concern [3]. The first evidence of the zoonotic transmission of HEV genotype 3 was found in Japan in 2003, when several cases of hepatitis E infection were linked to the consumption of pig and deer meat or organs [4, 5]. More case reports (grilled wild boar meat in Japan, pig meat in Spain, *figatelli* sausage from Corsica) have provided additional evidence that HEV is a zoonosis that can be transmitted via the consumption of contaminated food [6–8]. Admittedly, known viral RNA is an important marker of acute HEV infection, especially during early stages before the antibody response becomes evident [9]. However, until now, viral RNA has not been detected (in the representative sample) in Lithuanian pigs and wild ungulates such as wild boar, roe deer, red deer and moose. Thus, we aimed to gain insight through molecular investigation into HEV in these species as they are frequently used for human consumption. Furthermore, the availability of the generated HEV sequences may serve as a basis for interdisciplinary studies comparing human isolates to identify transmission interactions between animal and human hosts [10].

## Methods

The sample set for the study comprised 470 pig serum samples that had been collected randomly from farms by veterinarians within the framework of an official infectious disease surveillance program and 626 (n = 320 liver and n = 306 serum) samples from wild boar *(Sus scrofa*) (n = 505), roe deer (*Capreolus capreolus*) (n = 93), red deer (*Cervus elaphus)* (n = 15) and moose (*Alces alces*) (n = 13) that were hunted in 212 locations of Lithuania during the hunting seasons from 2014 to 2016.

Blood samples obtained from the wildlife were gathered from the heart or thoracic cavity into sterile plastic tubes. The serum was separated from the cellular elements by centrifuging the coagulated blood for 10 min at 2000×*g*. The extracted serum was stored at − 20 °C until further analysis. During the dressing of the carcasses, small pieces of hepatic tissues were also taken and stored at − 20 °C prior to further analysis.

### HEV RNA extraction and RT-PCR

Viral RNA was isolated from serum or liver samples with the Gene JET RNA Purification Kit (Thermo Fisher Scientific) according to the manufacturer’s recommendations. The extracted RNA was analyzed by nested reverse transcription polymerase chain reaction (RT-nPCR) using two HEV-specific sets of primers targeting the ORF1 and ORF2 fragments of the HEV genome (Table 1). The first amplification round was run in 25 µL of reaction mix containing 2.5 µL of extracted RNA, 12.5 µL of Dream Taq Green PCR Master mix (Thermo Fisher Scientific), 1 µL of the forward primer HEV-s (or 3156F), 1 µL of the reverse primer HEV-as (or 3157R), 0.3 µL of RevertAid Reverse Transcriptase (Thermo Fisher Scientific), 0.13 µL of RiboLock RNase Inhibitor (Thermo Fisher Scientific) and 7.12 µL of nuclease-free water (Thermo Fisher Scientific). The cycling conditions were: 42 °C for 30 min, initial denaturation at 95 °C for 5 min followed by 40 cycles of denaturation at 94 °C for 30 s (or 1 min if ORF2 primers were used), annealing at 50 °C for 30 s (or 60 °C for 1 min if ORF2 primers were used) and elongation at 72 °C for 45 s (or 1 min if ORF2 primers were used), followed by a final elongation at 72 °C for 10 min.

**Table 1:** Primer sets used in this study

Primer designation	Sequence (5ʹ → 3ʹ)	Step	Product length (bp)	Target region
HEV-s	TCGCGCATCACMTTYTTCCARAA	RT-PCR	469	ORF1
HEV-as	GCCATGTTCCAGACDGTRTTCCA			
HEV-fn	TGTTGCCCTGTTTGGCCCCTGGTTTAG	Nested RT-PCR	254	
HEV-rn	CCAGGCTCACCRGARTGYTTCTTCCA			
3156F	AATTATGCYCAGTAYCGRGTTG	RT-PCR	731	ORF2
3157R	CCCTTRTCYTGCTGMGCATTCTC			
3158Fn	GTWATGCTYTGCATWCATGGCT	Nested RT-PCR	348	
3159Rn	AGCCGACGAAATCAATTCTGTC			

Next, 2.5 µL of the product of the first amplification round was transferred to a new PCR mix containing 12.5 µL of Dream Taq Green PCR Master mix (Thermo Fisher Scientific), 1 µL of the forward primer HEV-fn (or 3158Fn), 1 µL of the reverse primer HEV-rn (or 3159Rn) and 8 µL of nuclease-free water (Thermo Fisher Scientific). The second-round cycling conditions were identical to those of the first except that the cycle at 42 °C for 30 min was not required and the annealing temperature of 50 °C was maintained for 30 s (or 55 °C for 1 min if ORF2 primers were used). All reactions were performed in a Mastercycler personal thermocycler (Eppendorf, Hamburg, Germany). The RT-nPCR products were separated on ethidium bromide-stained 1.8% agarose gels and visualized by UV light.

To minimize carryover, different parts of the process were physically separated from one another (in entirely separate working areas). A PCR hood and aerosol-barrier tips were used for the assembly of all reactions to avoid contamination. In every step, control reactions with no template were performed to check for contamination.

### Statistical analyses

Statistical analysis was conducted using the SPSS for Windows 15 statistics package (SPSS Inc., Chicago, IL, USA). The results were significant when P < 0.05. The descriptive data are presented as percentages. Fisher’s exact test was used to test for differences in prevalence of HEV and different target regions. HEV prevalence was calculated in pigs and wild animal species for the ORF1 and ORF2 sequences with 95% confidence intervals.

### Sequencing and phylogenetic analysis

The HEV-positive ORF2 RT-nPCR products were excised from the agarose gel, purified with a GeneJET PCR Purification kit (Thermo Fisher Scientific) and sequenced in both directions using the BigDye Terminator Cycle Sequencing kit v3.1 (Applied Biosystems) and the 3130 × Genetic Analyzer (Applied Biosystems). The sequences of both strands of the ORF2 PCR products were determined using the same primer set and identical cycling conditions as the nested PCR amplification. The sequences were submitted to GenBank.

The obtained ORF2 sequences (Accession Numbers MG739304–MG739318) were compared with the reference set of the selected sequences from GenBank, representing a full range of genetic diversity and geographic locations of the HEV genotype-3. The sequences were aligned using Clustal W software from MegAlign (Lasergene software package, DNASTAR Inc, Madison, USA). Bootstrap values were calculated using CLC Gene Free Workbench software, with bootstrap values based on 100 replicates (v4.0.01, CLC bio A/S, Aarhus, Denmark). Bootstrap values greater than 70% were considered to provide significant evidence for phylogenetic grouping.

## Results

The detailed HEV RNA results targeting different parts of the HEV genome are summarized in Table 2.

**Table 2:** Prevalence of HEV in domestic pigs and wild animal species using RT-nPCR assay

Investigated host	ORF1 targeting primers			ORF2 targeting primers		
	Sample type (number of HEV positive/tested samples (%))		All types of samples (number of HEV positive/tested (%, 95% CI))	Sample type (number of HEV positive/tested samples (%))		All types of samples(number of HEV positive/tested (%, 95% CI))
	Serum	Liver		Serum	Liver	
Domestic pigs (*Sus scrofa domesticus*)	155/470 (32.98)	–	155/470 (32.98%, 28.88–37.35)	106/470 (22.55)	–	106/470 (22.55%, 19.01–26.55)
Wild boars (*Sus scrofa*)	62/235 (26.38)	69/270 (25.56)	131/505 (25.94%, 22.31–29.93)	41/235 (17.44)	45/270 (16.67)	86/505 (17.03%, 14.00–20.55)
Roe deer (*Capreolus capreolus*)	10/45 (22.22)	11/48 (22.92)	21/93 (22.58%, 15.27–32.07)	7/45 (15.56)	5/48 (10.42)	12/93 (12.90%, 7.54–21.21)
Red deer (*Cervus elaphus*)	1/13 (7.69)	0/2 (0)	1/15 (6.67%, 1.19–29.82)	0/13 (0)	0/2 (0)	0/15 (0%, 0.00–20.39)
Moose (*Alces alces*)	1/13 (7.69)	–	1/13 (7.69%, 1.37–33.31)	0/13	–	0/13 (0%, 0.00–22.81)

In total, 155 of 470 (32.98%, 95% CI 28.88–37.35) and 106 of 470 (22.55%, 95% CI 19.01–26.55) domestic pig samples were positive for HEV RNA using RT-nPCR based on ORF1 and ORF2, respectively. The difference in positive detection rates between ORF1 and ORF2 was highly significant (P = 0.0004).

In wild animal species, 25.94% (95% CI 22.31–29.93) of wild boar samples, 22.58% of roe deer (95% CI 15.27–32.07) samples, 6.67% (95% CI 1.19–29.82) of red deer samples and 7.69% (95% CI 1.37–33.31) of moose samples were positive for HEV RNA using primers targeting ORF1. Viral RNA was detected in 17.03% (95% CI 14.00–20.55) of wild boar samples and 12.90% (95% CI 7.54–21.21) of roe deer samples targeting ORF2, while no HEV RNA was found in red deer or moose samples. Statistically significant differences in the proportion of the prevalence (%) detected by targeting the ORF1 and ORF2 fragments were observed for all investigated wild animal species except for roe deer.

**Fig. 1:**
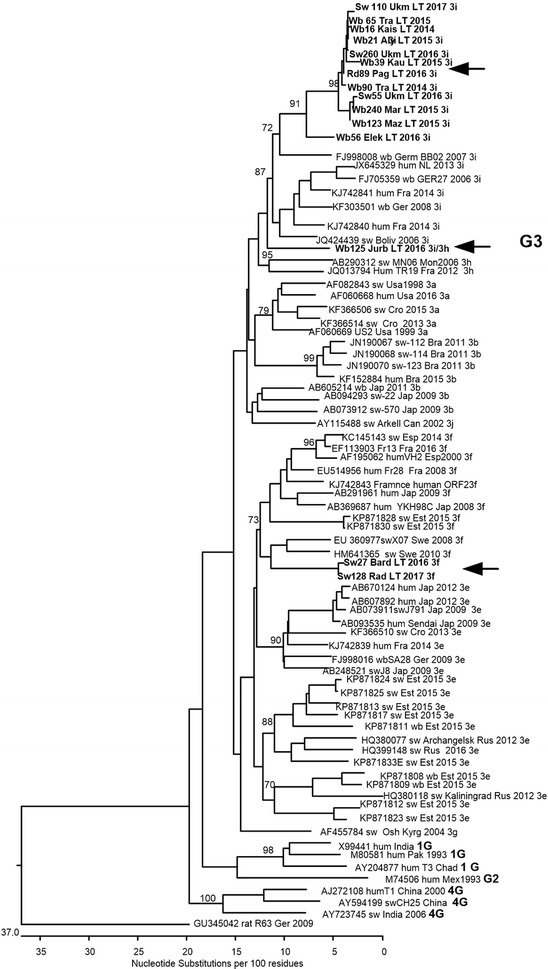
Phylogenetic analysis of Lithuanian HEV ORF2 sequences. Clustal W algorithm was used for sequence alignment. Numbers adjacent to main branches indicate bootstrap values for different genetic subtypes within HEV genotype3. The reference sequences are marked as follows: GenBank Accession Number, host and name of sequence, country (up to three letter abbreviations), year, subtype. The analysis involved 80-nucleotide partial HEV ORF2 sequences. Only bootstrap values > 70% are indicated. The sequences determined in this study (Accession Numbers MG739304–MG739318) are indicated in bold and with arrows

Samples from different hunting sites and pig farms were sequenced and analyzed to determine the HEV subtypes within different Lithuanian regions and hosts. Phylogenetic analyses based on a 348-nucleotide-long HEV ORF2 region showed that all obtained sequences detected in Lithuanian domestic pigs and wildlife belonged to genotype 3 (Fig. 1). Further subtyping was performed by comparing the obtained sequences with reference sequences representing the 3a, 3b, 3c, 3h, 3i, 3j subtypes of one major clade and the 3e, 3f, 3g subtypes of another major clade. The sequences identified in this study from pigs, wild boars and roe deer clustered within the 3i subtype reference sequences from the GenBank database, showing a homology of 88% (ranging from 86.8 to 88.9%). The 13 sequences from pigs, wild boars and roe deer clustered separately within subtype 3i, showing a mean homology of 96.3% (ranging from 96.3 to 100%). Two sequences from different pig farms clustered within subtype 3f sequences and revealed 85.3% (ranging from 71.6 to 99%) identity to reference strains of this HEV subtype. One HEV wild boar sequence clustered between subtypes 3i and 3h ORF2 reference sequences and exhibited 85.6–92.1% identity to subtype 3i and 87.6–86.4% to subtype 3h sequences.

## Discussion

The presence of HEV in food products derived from natural reservoirs of zoonotic HEV or food (fruits, vegetables, shellfish) that is contaminated by surface and irrigation water raises concerns for public health and food safety worldwide [11]. Autochthonous human HEV infections in industrialized countries (due to genotypes 3 and 4) are increasingly reported and are linked to zoonotic transmission, mainly through the consumption of contaminated meat and offal from pigs, Eurasian wild boar and deer that have been deemed to be plausible reservoirs for HEV [12]. Moreover, there is a category of meat from non-domesticated animals (game meat) that are hunted and slaughtered mostly for private consumption, but which can also be found in markets or restaurants. Although game meat represents only a small portion of the European market, its popularity as a luxury food source is growing worldwide. Wild boar and roe deer are the most common sources of game meat in Europe, including Lithuania, and have the largest harvest numbers [12, 13]. In addition, hunting, which is another recognized risk factor for zoonotic HEV transmission, is very common in Lithuania, with approximately 32 624 wild boars and 23 828 roe deer killed during the 2016–2017 hunting season [14]. Thus, the consumption of game meats and offal that may harbor HEV is as risky as eating pork [15].

To date, the detection of HEV is mainly carried out through qualitative or quantitative PCR. Extraction methods and detection protocols can vary significantly, and no gold standard approach has yet been defined for HEV diagnosis. The choice of primers used in RT-PCR assays varies from laboratory to laboratory. The differences in the sensitivity and specificity of various primers often lead to difficulties in comparing results from various studies. Therefore, caution must be taken when interpreting the results. It is known that standard RT-PCR is a sensitive technique, but its sensitivity can be markedly increased by performing nested RT-PCR. The nested strategy increases the specificity of RNA amplification by reducing the background due to non-specific amplification of RNA. Thus, for the direct screening of viral nucleic acids in samples and the ability to use the subsequent positive samples for genotyping, two different PCR assays were applied in this study. For the subtyping of HEV, we needed sequences in the ORF2 region that were 348 bp long. Many real-time RT-PCR products of ORF1, ORF2, and ORF3 are only 76–100 bp in length, which is not long enough for the molecular characterization of prevalent HEV strains.

Domestic pigs had a higher prevalence of HEV (22.55–32.97%) than wild ungulates. A possible reason is that the frequent direct contact among infected pigs reared in confined spaces may enhance the spread of HEV. Pigs housed in the same pen are exposed to the saliva, nasal secretions, urine, and feces of multiple pen mates repeatedly each day. Thus, the pig-farming environment may foster the spread of HEV among pigs compared to the environment of free ranging wild ungulates. The HEV RNA prevalence estimated in domestic pigs in the present study remains within the range found in other countries, such as Croatia (24.5%, [16]) and the USA (35%, [17]). Crossan et al. [18] reported HEV RNA in 44.4% of pig serum samples in Scotland, and Di Bartolo et al. [19] detected a viral prevalence of 64.6% in pigs in Italy, whereas Jori et al. [20] detected HEV RNA in only 8.3% of tested pig samples. The conducted studies have revealed that different viral prevalence exists among countries. This may reflect different infection dynamics related to farm-specific risk factors, such as farming scale, farming practices, biosecurity measures, and seasonal influence. [21].

The viral RNA prevalence in wild boars, roe deer, red deer and moose was 17.03–25.94%, 12.90–22.58%, 0–6.67%, and 0–7.69%, respectively. Despite the high densities of both wild boar and deer in Lithuania, a slightly lower HEV prevalence was observed in cervids (roe deer, red deer, moose) compared to that in wild boars, hinting towards interspecies transmission. Evidence suggests that deer may contract HEV from wild boars in cases where both species share the same habitat [12].

Other studies have identified HEV in 4.2% (24/566), 7.5% (8/106) and 12.3% of tested wild boars in Japan [22], the Netherlands [23] and Croatia [16], respectively. Our results agree with those reported by Mesquita et al. [24], where HEV RNA was detected in 25% (20/80) of the liver samples obtained from wild boars in Portugal. In contrast, the results from most studies of viral RNA prevalence varied widely even within the same country; HEV detection rates of 14.9% (22/148, [10]) and 68.2% (90/132, [25]) were found in wild boars in Germany, and 25% (22/88, [26]) and 0% (0/77, [27]) in Italy. Hence, the RNA detection method is crucial [19]. In fact, our results confirm different sensitivity with different targeted open reading frames, suggesting that the use of several RT-nPCR protocols may increase the sensitivity of HEV RNA detection [28]. The proportion of HEV RNA-positive samples for both open reading frames in this study did not significantly differ except between roe deer (22.58% vs. 12.90%, P = 0.084). However, the sensitivity of RT-PCR assays can vary widely, depending on target regions and HEV genotypes. Furthermore, sensitivity results might be affected by the quality of the RNA extraction procedure [29].

The prevalence of HEV infection among wild cervids has not yet been thoroughly investigated, and data are still inconsistent [3]. In Germany [30], 6.4% (5/78) of roe deer were positive for viral RNA, while an absence of HEV RNA was reported in the Netherlands (0/8) [23] and Sweden (0/29, 0/27) [31]. Our study results (ranging from 12.90 to 22.58% depending on the ORF fragment) are partially consistent with those of Forgach et al. [32], who found that 22% of roe deer (*Capreolus capreolus*) were positive for HEV RNA in Hungary.

In this study, 6.67 and 7.69% of red deer and moose samples were positive for HEV ORF1. The positive result could be caused by specific or unspecific amplification of ORF1 fragment and it is noted these animals HEV strains were not successful sequenced. Moreover, none of the 15 red deer or 13 moose samples were positive for the HEV ORF2 fragment. These results might be affected by a relatively small sample size. The reason for the absence of positive cases might be divergent HEV types that could not be detected by the assay used in this study [31]. Similar findings have been recently reported in Germany, where HEV was detected in 2.0–6.6% of red deer samples [33]. A higher HEV prevalence was noticed in red deer populations in Hungary (10%, [32]), Italy (11%, [34]) and the Netherlands (15%, [23]). There is a lack of surveillance data regarding the prevalence of HEV RNA in moose, which makes it difficult to compare prevalence trends. In another study, 15% of moose samples collected in 2012–2013 in Sweden were positive for viral RNA [35]. Similar results were reported by Roth et al. [31], who detected HEV in 11% (10/66) and 15% (7/11) of Swedish moose samples from 2012 to 2015.

Phylogenetic analyses of partial ORF2 HEV sequences have shown that several genetic subtypes of the HEV genotype 3 are present in Lithuanian pigs and wildlife [36]. The comparison of sequences obtained from wild boar, pig and roe deer samples showed a high degree of homology and clustered within subtype 3i reference sequences. This suggests that only the subtype 3i of HEV genotype 3 is circulating in Lithuanian pigs and wildlife. However, the clustering of the wild boar sequence (Wb125 Jurb LT 2016 3i/3h) between the 3i and 3h subtype sequences show that the wild boar population in Lithuania can also harbor additional subtypes of the HEV 3 genotype. This sequence showed 13.5–14% nucleotide variation compared to 3i subtype reference strains and 15–16% variation compared to subtype 3 h.

The HEV 3i subtype has been detected in Austria, Germany, France, Argentina, Bolivia, and Uruguay in various hosts, including humans [25, 36, 37], wild boars [10, 25] and domestic pigs [37, 38]. The ORF2 nucleotide sequence obtained in this study from roe deer (Rd89 Pag LT 2016 3i) shows that the HEV 3i subtype can be found in this species as well. Until recently, the 3i subtype had only been detected in wild boars in Germany, while in Austria and Argentina it has also been detected in humans [39]. The German wild boar strains of HEV sequences wbGER27 and BB02 were fully sequenced and used as reference HEV 3i subtype sequences [10, 25] in this study.

The sequences obtained from pig farms located in two different counties of Lithuania clustered with HEV strains from Estonia [40], Sweden [41], France [42], Croatia [16], and Hungary [32], and all of them were of the HEV 3f subtype. Interestingly, only one HEV 3f strain was isolated from wild boars, while all other 3f strains were isolated from humans to pigs. The presence of the HEV 3f subtype in Lithuanian pig farms may be due to import of animals from other parts of the EU as HEV strains isolated from wild boars and pigs in the neighboring regions of Estonia and the Kaliningrad district of the Russian Federation, have belonged to the 3e subtype [40].

## Conclusions

This study shows that pigs, wild boars, roe deer, red deer and moose in Lithuania may be infected with HEV. This calls for an increased public awareness of the zoonotic risk of HEV infection through food consumption or contact with infected animal populations.
