# Correction to: Prevalence and phylogenetic analysis of hepatitis E virus in pigs, wild boars, roe deer, red deer and moose in Lithuania

**DOI:** 10.1186/s13028-019-0443-7

**Published:** 2019-01-31

**Authors:** Ugne Spancerniene, Juozas Grigas, Jurate Buitkuviene, Judita Zymantiene, Vida Juozaitiene, Milda Stankeviciute, Dainius Razukevicius, Dainius Zienius, Arunas Stankevicius

**Affiliations:** 1 Department of Anatomy and Physiology, Faculty of Veterinary Medicine, Lithuanian University of Health Sciences, Tilzes str. 18, Kaunas, Lithuania; 2 National Food and Veterinary Risk Assessment Institute, J. Kairiukscio str. 10, Vilnius, Lithuania; 3 Department of Animal Breeding and Nutrition, Faculty of Animal Husbandry Technology, Lithuanian University of Health Sciences, Tilzes str. 18, Kaunas, Lithuania; 4 Faculty of Medicine, Lithuanian University of Health Sciences, A. Mickeviciaus str. 9, Kaunas, Lithuania; 5 Faculty of Veterinary Medicine, Institute of Microbiology and Virology, Lithuanian University of Health Sciences, Tilzes str. 18, Kaunas, Lithuania

## Correction to: Acta Vet Scand (2018) 60:13 10.1186/s13028-018-0367-7

Following publication of the original article [1], we have been notified of a typing error in the “Discussion” section.

The sentence “The proportion of HEV RNA-positive samples for both open reading frames in this study **did not significantly differ** except between roe deer (22.58% vs. 12.90%, P = 0.084).” should be read as: “The proportion of HEV RNA-positive samples for both open reading frames in this study **differed significantly** except between roe deer (22.58% vs. 12.90%, P = 0.084).”

## References

[1] Spancerniene U, Grigas J, Buitkuviene J, Zymantiene J, Juozaitiene V, Stankeviciute M, Razukevicius D, Zienius D, Stankevicius A (2018). Prevalence and phylogenetic analysis of hepatitis E virus in pigs, wild boars, roe deer, red deer and moose in Lithuania. Acta Vet Scand.

